# Defensive Driving: Directing HIV-1 Vaccine-Induced Humoral Immunity to the Mucosa with Chemokine Adjuvants

**DOI:** 10.1155/2018/3734207

**Published:** 2018-12-13

**Authors:** Ebony N. Gary, Michele A. Kutzler

**Affiliations:** ^1^The Department of Microbiology and Immunology, Drexel University College of Medicine, Philadelphia, PA, USA; ^2^The Division of Infectious Diseases and HIV Medicine, The Department of Medicine, Drexel University College of Medicine, Philadelphia, PA, USA

## Abstract

A myriad of pathogens gain access to the host via the mucosal route; thus, vaccinations that protect against mucosal pathogens are critical. Pathogens such as HIV, HSV, and influenza enter the host at mucosal sites such as the intestinal, urogenital, and respiratory tracts. All currently licensed vaccines mediate protection by inducing the production of antibodies which can limit pathogen replication at the site of infection. Unfortunately, parenteral vaccination rarely induces the production of an antigen-specific antibody at mucosal surfaces and thus relies on transudation of systemically generated antibody to mucosal surfaces to mediate protection. Mucosa-associated lymphoid tissues (MALTs) consist of a complex network of immune organs and tissues that orchestrate the interaction between the host, commensal microbes, and pathogens at these surfaces. This complexity necessitates strict control of the entry and exit of lymphocytes in the MALT. This control is mediated by chemoattractant chemokines or cytokines which recruit immune cells expressing the cognate receptors and adhesion molecules. Exploiting mucosal chemokine trafficking pathways to mobilize specific subsets of lymphocytes to mucosal tissues in the context of vaccination has improved immunogenicity and efficacy in preclinical models. This review describes the novel use of MALT chemokines as vaccine adjuvants. Specific attention will be placed upon the use of such adjuvants to enhance HIV-specific mucosal humoral immunity in the context of prophylactic vaccination.

## 1. Introduction

Many pathogens access the host via mucosal barrier surfaces. Thus, developing vaccines that elicit robust effector and memory responses at mucosal sites is a crucial public health goal. The mucosa-associated lymphoid tissues (MALTs) are an interactive network of organs and tissues that are responsible for the education of mucosal lymphocytes and the orchestration of responses against commensal microbes and pathogens. As the mucosal immune system must balance the ability to respond to pathogens with tolerance of commensal microbes, effector cell access to the MALT is tightly regulated. Peripherally activated lymphocytes are rarely able to traffic to mucosal sites due to low, or lack of expression, specific adhesion and chemokine receptors required for entry into these sites. Due to the exclusion of these peripheral lymphocytes, generating mucosal immunity with parenteral vaccination is rarely successful. While it has been demonstrated that peripheral vaccination can generate mucosal humoral responses, it does so by relying on the magnitude of the response. Vaccinating with adjuvants in the periphery induces large quantities of antigen-specific antibodies. This increased concentration of the antigen-specific antibody can then transudate to mucosal surfaces. Thus, even in the context of peripheral vaccination, successful mucosal targeting of responses has the potential to have dose-sparing a effects on vaccine development.

Before the discovery of mucosa-specific chemokines, it was known that a common mucosal immune system existed. Czerkinsky et al. and Bienenstock et al. reported that following adoptive transfer, labeled antibody-secreting cells (ASCs) from mesenteric lymph nodes (MLNs) of donor mice were more likely to be recovered from the intestines, mammary glands, cervix, vagina, and MLN of recipient mice [1–3]. These data supported the idea that mucosal immunity is a coordinated phenomenon, namely, that there are cell-intrinsic differences in the ability of lymphocytes to access the MALT. Subsequent studies in mice and other animal models confirmed the existence of the common mucosal immune system [4]. We now know that access to the MALT is dependent upon the expression of specific chemokine receptors. Chemokines are small 8–14 kD secretory proteins classified by the arrangement of four canonical cysteines into four classes—the CXC or alpha chemokines, the CC or beta chemokines, the C or gamma, and the CX3C or delta chemokines. The cell-expressed G-protein chemokine receptors that bind them are similarly classified [5]. Directing immune responses to the mucosa remains a challenge for HIV vaccine design. As human immunodeficiency virus-1 (HIV-1) is primarily transmitted sexually, with infection occurring in the gastrointestinal and genital mucosae, the induction of robust humoral responses in the mucosa is critical to the development of an efficacious prophylactic vaccine. Harnessing the extant chemokine/receptor system responsible for trafficking antibody-secreting cells to mucosal surfaces during and after immunization is a viable strategy for enhancing antigen-specific immunity in the mucosa. Here, we discuss HIV-1 infection in the mucosa, and the necessity and challenges of designing an HIV-1 vaccine. We will also discuss the chemokines and receptors responsible for mucosal trafficking of lymphocytes and review recent studies using chemokines to augment mucosal responses to viral vaccine antigens including HIV, HSV, and influenza.

### 1.1. Mucosal Pathogenesis of HIV

Human immunodeficiency virus-1 (HIV-1) currently infects more than thirty-five million people, and the WHO estimates that 0.8% of all adults between ages 15 and 50 are HIV infected. More than 2.5 million new infections occur each year, highlighting the need for an effective prophylactic vaccine. Unfortunately, the nature of the virus lifecycle and the lack of definitive correlates of protection make vaccine design challenging [6–8]. HIV-1 transmission occurs primarily through sexual contact, at mucosal surfaces. Once the virus accesses tissue resident CD4^+^T cells, its primary targets, integration of the viral genome into the host genome establishes lifelong infection. An effective prophylactic vaccine must therefore engender a robust, neutralizing antibody response directed specifically to the mucosal compartment. Directing vaccine-induced responses to mucosal sites remains remarkably challenging. The concentration of antigen-specific antibody in the mucosal compartment following immunization depends on several factors, namely, the dose of antigen and the route of delivery. It is generally known that oral vaccination is the best delivery route for generation of mucosal antibody responses; unfortunately, oral antigen delivery requires large doses to overcome the tolerogenic environment of the gastrointestinal tract. Similarly, direct delivery of the antigen to mucosal sites, such as intranasal administration, while effective, requires large amounts of the antigen to mediate immunity. Conversely, relatively small doses of the protein antigen delivered parenterally induce robust IgG responses, including neutralizing antibody production, but little to no mucosal antibody of either IgG or IgA isotypes. Increasing dosages of the protein antigen and the addition of adjuvants can promote high titers of the antibody that can passively diffuse into the mucosa, leading to protection against infection.

Despite the substantial number of HIV-1 vaccine clinical trials completed and underway, almost no successes have been recorded. The only trial to ever demonstrate efficacy was the RV144 or Thai trial, which began in 2003 [9]. The vaccine regimen consisted of a modified canarypox vector expressing HIV-1 *gag*, *pol*, and *env* proteins, followed by a recombinant HIV gp120 (envelope, *env*) boost. The trial demonstrated efficacy ranging from 26.4%–31%. It was determined after analysis of patient samples that protection correlated with HIV-specific serum IgG which mediated antibody-dependent cellular cytotoxicity (ADCC). This indicated that nonneutralizing antibodies (nNAbs) may play an important role in preventing HIV infection (for a review of nNAbs in HIV, see Excler et al. [10]). Interestingly, subsequent analysis of serum from RV144 vaccinees determined that while env-binding IgG inversely correlated with risk of infection, env-binding IgA in serum positively correlated with infection risk [9]. For in-depth review of the analysis of correlates of protection in this trial, see Kim et al. [8]. It should be noted that mucosal IgA is mostly found in dimeric form (dIgA) and serum IgA is mostly found as monomeric IgA [11]. Unfortunately, no mucosal samples were taken from RV144 vaccinees; thus, the effect of mucosal dIgA on transmission could not be determined in subsequent analyses [12].

Very few studies have evaluated the ability of neutralizing antibodies of the IgA isotype to prevent infection. In one such study, Watkins and colleagues found that intrarectal application of dimeric IgA1 (dIgA1) to rhesus macaques prior to intrarectal challenge with simian-human immunodeficiency virus (SHIV) led to 83% protection from challenge [13]. While analysis from RV144 trial vaccinees indicated that serum IgA positively correlated with infection risk, Sholukh and colleagues found that the combination of dIgA and IgG (targeting the same HGN194 (a neutralizing epitope in env)), applied intrarectally, led to 100% protection from intrarectal challenge [14]. These results suggest different roles for serum IgA and mucosal dIgA. Recently, to understand the role of vaccine-induced IgA, HIV-specific IgA monoclonal antibodies (MAbs) were cloned from memory B cells present in the blood of RV144 vaccinees. These HIV-specific IgA MAbs were capable of mediating antibody-dependent cellular phagocytosis (ADCP) by monocytes and blocked env-binding to the alternative HIV receptor galactosylceramide [15]. In support of this, several studies have determined that there is an association of HIV-specific mucosal IgA with reduction of infection. Decreased risk of mother to child transmission was associated with anti-env IgA in breast milk [16]. Similarly, decreased infection rates in exposed seronegative women were associated with HIV-1 neutralizing IgA in genital secretions [17]. Finally, neutralizing mucosal IgA was detected in a cohort of exposed, seronegative Kenyan sex workers [18]. These data indicate that both neutralizing and nonneutralizing IgA antibodies at mucosal surfaces may be an important correlate of HIV-1 protection. The potential role of mucosal IgA in mediating protection from HIV infection necessitates a clear understanding of the function of inductive and effector mucosal immune organs, as IgA-secreting B cells are induced and educated at these sites.

## 2. The Mucosal Immune System

**Figure 1:**
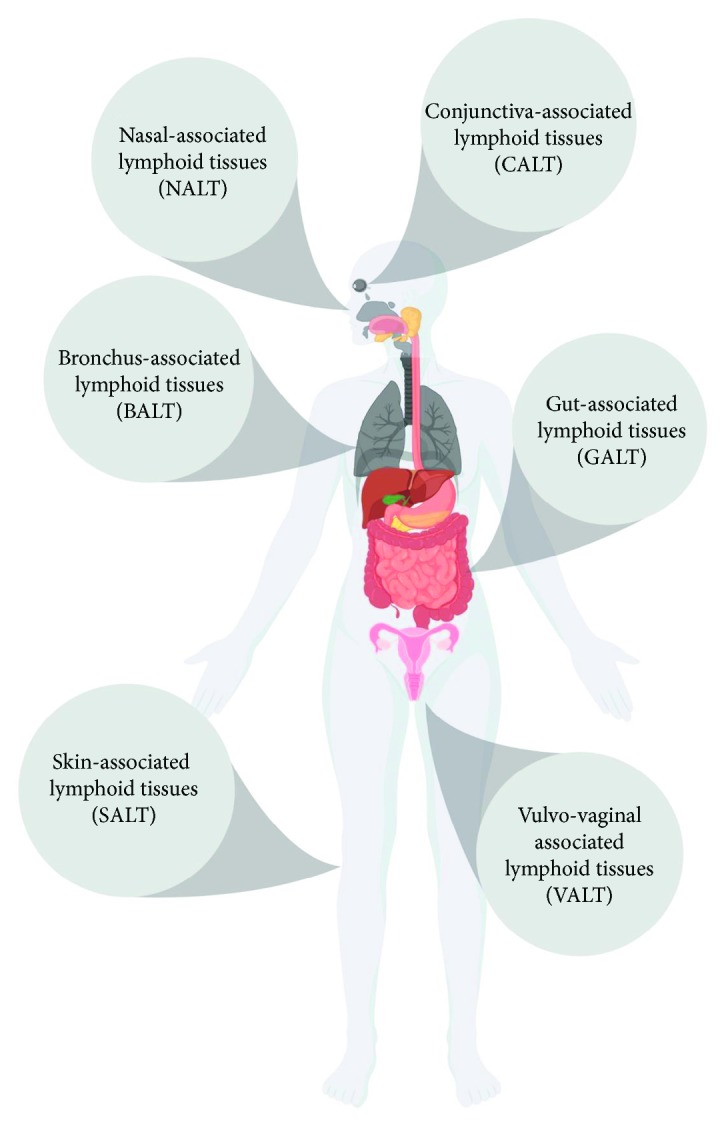
Mucosa-associated lymphoid tissues. MALT provides protection from pathogenic incursion and promotes the development of tolerance to commensal microbes. The lymphoid tissues in these sites sample the antigen directly from the environment to initiate immune exclusion or immune tolerance, and these responses are propagated in associated draining lymph nodes.

Mucosal surfaces present a barrier between the host and the environment and must balance tolerogenic responses to commensal microbes while maintaining the ability to respond to pathogens. The mucosal immune system consists of lymph nodes and nonorganized lymphoid tissues present in the respiratory, digestive, ocular, mammary, and urogenital tracts. There is also evidence of a connection between the skin immune system and the classical mucosal immune system [19, 20] (Figure 1—MALTs). IgA is found at high concentration in mucosal sites (where IgG is also present in substantial quantities) and at low concentration in the serum. In humans, two isotypes of IgA exist, IgA1 and IgA2 [21]. Interestingly, in the female genitourinary tract, antibodies of the IgG isotype are found in greater quantities than IgA [22]. The regulation of production and secretion of IgA is a key component of the mucosal barrier system. Differences in affinity of secretory IgA (sIgA) can determine if an antigen is subject to immune protection or tolerance [23–26]. Low-affinity binding to commensals is proposed to induce immune exclusion or tolerance to these bacteria, and this is required for the development and homeostasis of the mucosal immune system. Conversely, high-affinity IgA is proposed to bind pathogens and subject them to immune control.

The gut-associated lymphoid tissues (GALTs) are the largest lymphoid tissue organization in mammals. It consists of discrete organs: Peyer's patches (PPs), appendix, and isolated lymphoid follicles (ILFs). The GALT also contains diffuse lymphoid tissues including intraepithelial lymphocytes (IELs) and lamina propria (LP) lymphocytes. The appendix, ILFs, and PPs are considered inductive sites of mucosal immunity, while the MLNs and LP are considered effector sites. The GALT is part of the MALT (which encompasses all mucosa-associated lymphoid tissues) but distinct from the nasopharynx-associated lymphoid tissues (NALTs) which begin in the upper palate and include the nasal and upper respiratory tract mucosa. As IgA^+^ B cells leave inductive sites in the MALT, they terminally differentiate to plasma cells, resulting in a greater number of IgA-secreting B cells in mucosal effector sites (MLNs and LP) than in inductive sites (PPs and appendix) [27]. This phenomenon is termed the “IgA cycle” [28] and is supported by genomics studies linking the immunoglobulin variable heavy (Ig_v_H) chains of PP B cells to those of LP B cells [29–31]. Mesenteric lymph nodes are considered a part of the MALT, as activated mucosal lymphocytes can drain here and undergo expansion, but some researchers have suggested that they cannot be included in the MALT as the MALT proper samples antigen directly from intestinal lumen [32]. It is important to note that like the PPs, all MALT organs/organelles are similar in structure to peripheral lymph nodes, with discreet B cell zones separated by T cell areas, and contain dendritic cells and other antigen-presenting cells. Importantly, MALT organs and organelles lack afferent lymphatics. This lack of afferent lymphatics is possible as the characteristic follicle-associated epithelium (FAE) of the MALT contains microfold or M cells, which directly sample luminal antigens [33], and only efferent lymphatics are required for activated cells to exit and access other sites.

**Figure 2:**
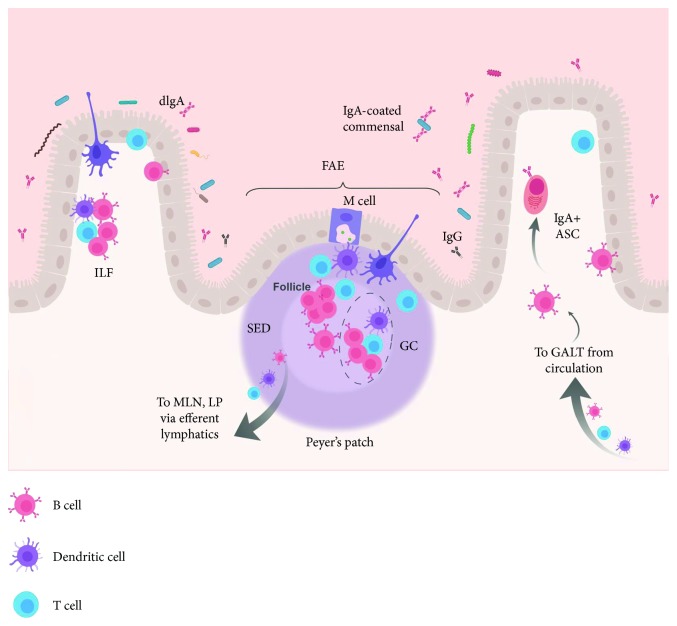
The Peyer's patch (PP) is the inductive site of GALT responses. M cells in the follicle-associated epithelium (FAE) of the PP directly sample luminal antigens and deliver them to antigen-presenting cells in the basolateral tissue. Dendritic cells (purple) may also stretch across the FAE and sample antigen directly. Antigen is presented to T cells (blue) within the PP. CD4^+^ T cells (blue) provide help to PP B cells (red). Within the subepithelial zone (SED), PP germinal centers (GC), T_FH_ induce T-dependent IgA class switching of BCRs. Similarly, isolated lymphoid follicles (ILFs) are also inductive sites of MALT responses. Activated lymphocytes can exit the PP via efferent lymphatics and traffic to the mesenteric lymph node (MLN) or lamina proria (LP), and return to the GALT from circulation. In the GALT, antibody-secreting cells (ASCs) secrete antibodies including dimeric IgA (dIgA) which are translocated into the lumen.

Peyer's patches are the primary inductive sites of IgA responses in the intestine [34, 35]. These patches are small, domed structures, visible to the naked eye, containing lymphocytes including B cells, T cells, and dendritic cells. In mice, the small intestines contain 7–12 PPs along its length. In humans, the number ranges from 30 to more than 200 [36]. Peyer's patches have distinct anatomical regions. B cell zones or follicles are surrounded by a FAE. The subepithelial dome (SED) lies between the FAE and the B cell follicles. Small T cell zones are also present in the PP [37] (see ref. [29] for a complete review of PP biology). The FAE microfold cells (M cells) sample luminal antigens and present them in Peyer's patches [38]. Germinal centers (GCs) are formed in the SED where follicular helper T cells (T_FH_) induce T-dependent IgA class switching in B cells [37, 39] (Figure 2—Peyer's patch). The size and complexity of the mucosal immune system and the crosstalk between individual units of the MALT present challenges for vaccine design. However, the concept of a “unified” mucosal immune system implies that vaccine modalities that enhance mucosal responses will produce effects in multiple mucosal sites. This is especially helpful in the context of HIV vaccines as HIV transmission occurs primarily at gastrointestinal and urogenital mucosal sites.

## 3. Mucosal Chemokines and Their Receptors

**Figure 3:**
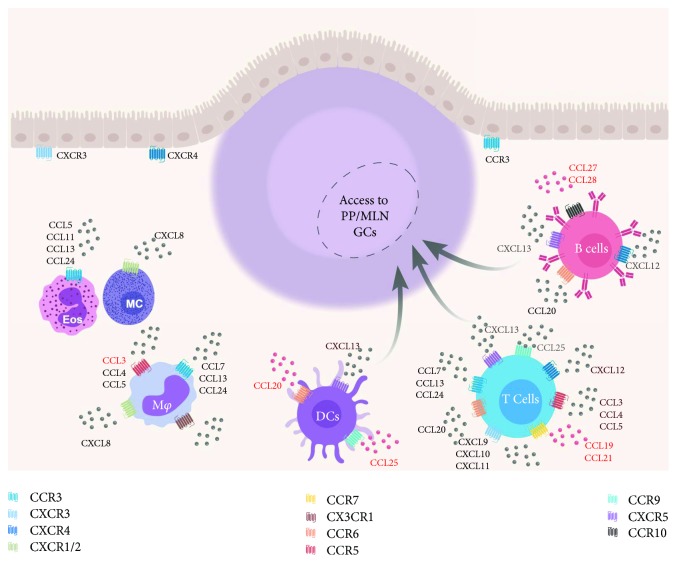
GALT chemokines and their receptors. All currently known GALT chemokines and their associated receptors are depicted. Chemokines and receptors are separated by the cell type. Chemokine adjuvants that have been used in the context of vaccination are depicted in red.

The intestinal epithelial lining is dynamic and mediates interactions between the environment and the host. Intestinal epithelial cells (IECs) and the immune cells which reside in the tissue beneath the epithelial layer are responsible for maintaining the balance between responding to pathogens and tolerance of commensals. IECs express more than twenty unique chemokines, which bind ten distinct receptors (see Kulkarni et al. for a review of chemokines expressed by IECs [40]) (Figure 3—chemokine trafficking in the mucosa). The CXC chemokines CXCL8, 9, 10, 11, 12, and CXCL13 are expressed in the MALT. CXCL8 binds the receptors CXCR1 and CXCR2 expressed on eosinophils [41], mast cells [42], neutrophils [43], and some macrophages [44]. CXCL9, 10, and 11 all bind the receptor CXCR3 expressed on T_H_1 cells [45]. CXCL12 binds the receptor CXCR4 which is expressed on IgA^+^ and IgG^+^ plasma cells [46, 47], and T cells (and is also a coreceptor for HIV infection). CXCL13 is expressed in peripheral and mucosal secondary lymphoid organs and grants B cells, T cells, and dendritic cells access to GCs via expression of the receptor CXCR5 [48]. The GC is the primary site of T-dependent class switch and affinity maturation [49, 50]. PP T_FH_ are most likely to induce IgA class switching. Importantly, in the context of HIV-1 infection, T_FH_ cells residing within lymphoid tissue GCs are a reservoir of infection-competent virus [51–54]. The CX3C chemokine CX3CL1 (fractalkine) binds the receptor CX3CR1, which is expressed on macrophages and dendritic cells [55, 56].

A variety of CC chemokines are also expressed by MALT IECs. The CC chemokine receptor CCR3 is expressed on eosinophils [57], macrophages [58], and T cells [59] and binds the chemokines CCL5, CCL7, CCL11, CCL13, CCL24, and CCL24, all of which are expressed by IECs. CCR5 is expressed on monocytes, macrophages [60], and T cells [61] and binds the ligands CCL3, CCL4, and CCL5. Importantly, CCR5 and CCR3 [62–64] to a lesser extent, along with the chemokine receptor CXCR4, are known coreceptors for HIV-1 infection. CCR6, an important mucosal homing receptor is expressed on dendritic cells, mature B cells, and T cells including T_H_17 cells. CCR6 has only one known ligand, CCL20 [65]. CCR7 which is expressed by activated T cells [66] as well as innate lymphoid type 3 cells (ILC3) [66, 67] binds both CCL19 and CCL21.

The three most well-studied mucosal chemokines are CCL25, CCL27, and CCL28. The chemokine CCL25 (also called thymus-expressed chemokine or TECK), a well-studied skin-homing chemokine, has only one receptor, CCR9, and attracts gamma-delta T cells (*γδ*T) [68], CD8^+^ T cells [69], CD4^+^ T cells [70], dendritic cells [71], and IgA^+^ plasma cells [72] to the MALT [73]. The CCR9/CCL25 axis is associated with oral tolerance [74], and perturbations in this axis are associated with pathogenic inflammation [75, 76]. CCL28 or mucosa-associated epithelial chemokine (MEC) is secreted by epithelial cells at many mucosal surfaces including the colon, salivary glands, mammary glands, and respiratory and urogenital tracts [77, 78]. CCL28 binds the receptor CCR10 and was first described by Mora et al. and Wang and colleagues [20, 79]. CCL28 is the most well-studied mucosal chemokine and is associated almost exclusively with the homing of IgA^+^ antibody-secreting cells [73, 80–84]. Both CCL25, CCL28, and their receptors are expressed early in gestation in the thymus and mucosal tissues, suggesting involvement in the ontogeny of the common mucosal immune system [85]. The second ligand of CCR10 is CCL27 (also called cutaneous T cell-attracting chemokine or CTACK) [86] which is associated most commonly with the homing of T lymphocytes to the skin [87], but is indeed expressed by IECs.

The expression of the various receptors is “imprinted” on naïve lymphocytes following antigen stimulation. In the case of T cells, the antigen presented by mucosal dendritic (CD103^+^) cells leads to the upregulation of CCR9 and the adhesion molecule *α*4*β*7 which binds MALT-expressed mucosal vascular addressin cell adhesion molecule 1 (MADCAM1). For B cells, the expression of CCR9, CCR10, and *α*4*β*7 provides access to the MALT. The end results of these complex interactions and receptor profiles are the attachment and extravasation of all the major types of lymphocytes into the MALT.

HIV transmission occurs primarily in the mucosa, and these surfaces are the sites of initial virus replication before dissemination and latency. Much research has therefore been focused on the role of chemokines and inflammatory cytokines at mucosal surfaces and HIV susceptibility or resistance. Recently, Arnold et al. demonstrated a striking correlation between inflammatory chemokines, decreased mucosal barrier integrity, and susceptibility to HIV infection [88]. The recruitment of CD4^+^ T cells and other infection-permissive cells increases the number of target cells and can enhance HIV infection at the mucosa. Interestingly, increased levels of HIV coreceptor-binding mucosal chemokines CCL3 [89] and CCL5 [90] have been associated with decreased susceptibility to HIV infection. This is thought to be due to competition for coreceptor binding. Elevated levels of the mucosal chemokine CCL20 in cervicovaginal wash from HIV-infected and uninfected women correlated with inhibition of HIV infection *in vitro* [91]. As the receptor for CCL20, CCR6, is not a known HIV coreceptor, it has been suggested that CCL20 might have anti-HIV antimicrobial activity. It was subsequently reported that the CCR6/CCL20 interaction stimulates cell-intrinsic immunity via cellular restriction factors [92]. For a complete review of barrier chemokines and their role in HIV pathogenesis, see Rancez et al. [93]. These studies strongly indicate a role for chemokine/receptor signaling in HIV infection, pathogenesis, and resistance. This supports the hypothesis that the chemokine trafficking system of the MALT could be strategically employed to prevent HIV infection.

## 4. Chemokine Adjuvants for Antiviral Mucosal Vaccines

The recruitment of activated lymphocytes to mucosal surfaces is strictly controlled, requiring the expression of specific chemokine receptors and adhesion molecules. This selection helps prevent pathogenic mucosal inflammation but presents a challenge for parenteral vaccination. Herpes virus infections are typically transmitted at mucosal surfaces. In the context of herpes infection, CD8^+^ T cell responses are critical to protection; however, neutralizing antibodies can also prevent transmission. In a landmark publication, Shin and Iwasaki proposed the topical application of chemokines to “pull” antigen-experienced T lymphocytes that had been primed by peripheral vaccination, to the vaginal tract [94]. They called this approach “prime and pull,” and it was remarkably effective. Following subcutaneous immunization with an attenuated herpes virus type-2 (HSV-2) and a topical application of the T cell chemokines CXCL9 and CXCL10 (CXCR3 ligands—CXCR3L) in the vaginal tract, HSV-2 glycoprotein B (gB)-specific CD8^+^ T cells were detected in the vaginal mucosa. Specifically, the detected cells were CXCR3-expressing and had an activated phenotype. Importantly, these cells remained in the vaginal mucosa for up to twelve weeks post-“pull,” and this led to 100% protection from lethal vaginal challenge [94]. The prime and pull approach definitively proved that the mucosal chemokine system could be used to direct antigen-specific responses to mucosal surfaces.

There are technical and logistical challenges associated with producing recombinant chemokines and delivering them to the genital mucosa in human patients. The DNA vaccine platform solves many of these technical issues. Delivering DNA-encoded chemokines peripherally would enhance MALT receptor expression on antigen-specific cells, enabling them to traffic to the mucosa more effectively, bypassing the need for direct delivery of the chemokine to the genital mucosa. DNA vaccines typically consist of naked plasmids encoding the DNA sequence of the protein of interest. Upon delivery, this DNA is taken up by cells, transcribed and translated within the cell, processed and presented on MHC molecules, and secreted as a soluble antigen from transformed cells [95]. DNA is incredibly stable, can be synthesized in the lab, and requires no cold chain transport. Finally, the advent of electroporation for delivery and the optimization of plasmid generation has enhanced the immunogenicity of DNA vaccines [96, 97]. The DNA platform also allows for the inclusion of plasmid-encoded adjuvants, termed “molecular adjuvants,” such as chemokines, to be codelivered with antigens in a single formulation. Importantly, DNA vaccines have been used in humans for over two decades and have an excellent safety profile [98].

Herpes DNA vaccines have capitalized on the flexibility of the DNA platform to deliver HSV antigens and mucosa-directing chemokines to target vaccine responses to the genital tract. In 2001, when comparing intramuscular versus intranasal vaccine delivery, Eo and colleagues reported that intranasal codelivery of plasmids encoding Herpes gB and murine CCL19 and CCL21 leads to a transient increase in HSV-specific IgA in vaginal wash, while intramuscular immunization did not enhance mucosal antibody [99]. Similarly, the Rouse lab demonstrated an increase in vaginal IgA in response to a viral vector prime, the DNA boost vaccine regimen. The formulation included plasmid-expressed gB, CCL21 or CCL19, and recombinant vaccinia virus, encoding herpes gB. Unfortunately, these responses did not lead to the generation of long-lasting memory [100].

Using a slightly modified approach, Yan and colleagues created a fusion plasmid encoding both HSV-2 gB and CCL19 and injected 5 *μ*g of this single plasmid into female mice twice, separated by two weeks. The animals were rested for seven weeks and then given a lethal intravaginal challenge with HSV-2. The fusion construct was superior to immunization with separate plasmids encoding gB and CCL19. However, immunization with either the fusion construct or two individual plasmids leads to statistically significant increases in serum and vaginal HSV-specific IgA and serum IgG. The group also observed increases in IgA-secreting cells in the colorectal mucosa and enhancement of spleen and MLN lymphocyte migrations toward CCL19, indicating increased expression of the receptor CCR7 and explaining the increased mucosal antibody responses. These enhanced antibody responses lead to protection from challenge. Animals immunized with either the two plasmids or the single fusion plasmid had decreased mortality; however, animals receiving the individual plasmids lost weight and had mild, clinical disease, while those immunized with the fusion construct lost no weight and exhibited no clinical disease [101]. This indicates a benefit of having the chemokine adjuvant and antigen expressed in the same transformed cell.

Influenza vaccines face similar challenges in that flu-specific immunity needs to be present in the upper respiratory mucosa to protect against viral infection. In the context of influenza, neutralizing antibodies at the site of transmission are critical to preventing infection. CD8^+^ T cell responses are equally critical in that the recognition and killing of influenza-infected cells will limit replication and protect against disease. Traditional intramuscular influenza vaccines with protein antigens and chemical adjuvants induce robust peripheral responses, generating high titers of neutralizing IgG which can diffuse form circulation into the BALT and NALT. Our laboratory evaluated the use of CCL27 and CCL28 to augment responses to an influenza hemagglutinin- (HA-) encoding DNA vaccine [102]. This was the first use of these chemokines as molecular (plasmid-encoded) adjuvants in the context of a DNA vaccine. We observed 2–3-fold increases in HA-specific IgA in the fecal extract of vaccinated animals, which remained detectable at 8 weeks after a second intramuscular immunization. The presence of antigen-specific antibodies at distal mucosal sites is indicative of coordinated mucosal homing. That peripherally activated lymphocytes can traffic to the mucosa following chemokine-adjuvanted vaccination suggests that these chemokine molecules imprint such cells with the receptors necessary for mucosal trafficking. We also detected increased IgG in the serum of chemokine coimmunized animals. This neutralizing IgG led to 100% protection from lethal influenza infection in these animals [102]. The Kutzler laboratory has also used CCL25 (TECK) to enhance influenza-specific T cell responses at mucosal surfaces. Again, we detected increased HA-specific IgA in the fecal extracts and vaginal wash from CCL25 coimmunized animals as well as increased IgA ASCs in the lungs of these animals. Importantly, increased IFN*γ*-secreting T cells in the spleens and MLNs of these animals were also evident. Upon challenge, pCCL25 and pHA coimmunized animals lost less weight and were 100% protected from mortality [103].

Creating an effective anti-HIV vaccine requires the generation of effective humoral and cell-mediated responses. Binding, neutralizing, and ADCC/ADCP-mediating antibody responses are as critical as the generation of effector CD8^+^ T cells. Importantly, the vast majority of these responses need to be directed to mucosal surfaces to prevent transmission of the virus. In keeping with this, HIV vaccine researchers have used mucosal chemokines to enhance B and T cell responses to HIV-1 immunogens. Song and colleagues reported that immunization with 50 *μ*g of HIV gag (capsid proteins) plasmid in the presence of CCL3, CCL19, and CCL20 leads to enhanced recruitment of macrophages and CD8^+^ T cells. Unfortunately, B cell activation was lacking, and only a modest enhancement in HIV-specific IgG was reported in animals receiving pgag and pCCL19 [104].

CCL25, CCL27, and CCL28 are some of the most well-studied mucosal chemokines and are used to promote the generation of antigen-specific mucosal immunity following immunization. We reported on the use of CCL25 to drive T cell responses to the mucosa following HIV DNA immunization, finding that after immunization via electroporation, increased IFN*γ*-secreting cells were detected in the spleen and MLN of coimmunized animals, and increased HIV-specific IgA in the serum and fecal extracts was also detected in these mice [103]. Our laboratory was the first to report the use of CCL27 and CCL28 as molecular adjuvants in the context of a DNA vaccine [102]. Other groups have confirmed our findings and used these chemokines in the context of HIV-1 vaccines. Hu et al. completed a comparative study of the ability of chemokine and cytokine adjuvants to augment an HIV-1 env gp140 DNA vaccine when delivered either intramuscularly with electroporation or intranasally as naked DNA. The group vaccinated mice with either pgp140 alone, plasmid-encoded CCL19 and CCL28, or a proliferation-inducing ligand (APRIL, a known B cell-stimulating cytokine). The results demonstrated that coimmunization with pCCL19 or pCCL28 enhanced mucosal and systemic anti-HIV IgA responses. Importantly, neutralizing IgA from vaginal secretions was reported in this study. Finally, Hu and colleagues reported detecting increased CCR10^+^ B cells in the MLNs of CCL28 coimmunized animals when the vaccine was delivered intramuscularly with electroporation but not when delivered intranasally [105]. These results also demonstrated that expression of the associated chemokine receptor is required for chemokine adjuvanticity. In this study, 15 *μ*g of chemokine adjuvant was delivered with 4 *μ*g of the env antigen in PEI (transfection reagent) intranasally, while 30 *μ*g of the antigen and 100 *μ*g of adjuvant were used for intramuscular delivery via electroporation. Intranasal delivery may have been less successful in these studies due to the decreased concentration of the antigen and adjuvant used. These results however, indicate that CCR10 expression mediated CCL28-induced responses.

In an attempt to replicate the Iwaski “prime and pull” method of classical protein immunization and topical application of chemokine in the context of an HIV-1 vaccine, Tregoning and colleagues vaccinated animals with trimeric HIV-1 gp140 and applied CCL28 to the vaginal surface six days after each immunization. They detected an increase in total, but not HIV-specific IgA in the vaginal wash of immunized animals [106]. The group did not examine CCR10 expression on the surface of IgA-secreting cells in this study. These results further support the need for expression of chemokine and antigen together during priming as being critical for enhancing mucosal homing of antigen-specific cells.

Following the encouraging results reported by our laboratory and others using CCL28 to enhance HIV-specific IgA responses in the mucosal tract, we performed similar experiments in nonhuman primates. Female macaques were vaccinated five times, separated by 6 weeks, with plasmids encoding consensus simian immunodeficiency virus (SIV—the NHP analogue of HIV) gag, pol, and env, followed by a boost with SIV nef-rev plasmids. HIV antigens were administered either alone or with plasmids encoding rhesus CCL25 (CCR9L) or CCL27 and CCL28 (CCR10L). All immunizations were given by intramuscular injection followed by electroporation. In these studies, we detected increased mucosal and systemic IgG and IgA in coimmunized animals. The primates which received CCR10L-encoding plasmids had an 89% protection rate from SIV challenge compared to only 68% protection in the other vaccine groups and 14% in naïve primates [107]. These increased antibody responses were correlated with a decrease risk of infection during challenge. Our studies and those performed in other laboratories have demonstrated the ability of CCL27 and CCL28 to enhance mucosal IgA responses to HIV vaccines, promoting increased antigen-specific IgA in the mucosal secretions of animals, which can mediate transmission prevention (Table 1—chemokine vaccine studies targeting HIV). Importantly, all the studies described above demonstrate that peripheral immunization, using molecules that target antigen-specific cells to the mucosa, can induce mucosal immunity.

## 5. Discussion

The characterization of the chemokines and receptors involved in the tissue-specific migration of immune cells has yielded a greater understanding of how vaccine adjuvants can be used to target antigen-specific immunity to the mucosa. This understanding will be crucial to the development of vaccines against mucosal pathogens. Poor mucosal responsiveness to parenterally delivered vaccine antigens highlights the need to develop vaccine modalities that direct antigen-specific cells to barrier surfaces. There is an urgent need to develop a safe, immunogenic HIV-1 vaccine that generates binding and neutralizing antibodies, effector T cells, and promotes the formation of long-lasting memory at mucosal surfaces. The challenges associated with HIV vaccine development, namely, the lack of clear correlates of protection, make this difficult. However, immunity at the genital mucosa will obviously play a role in preventing transmission.

**Figure 4:**
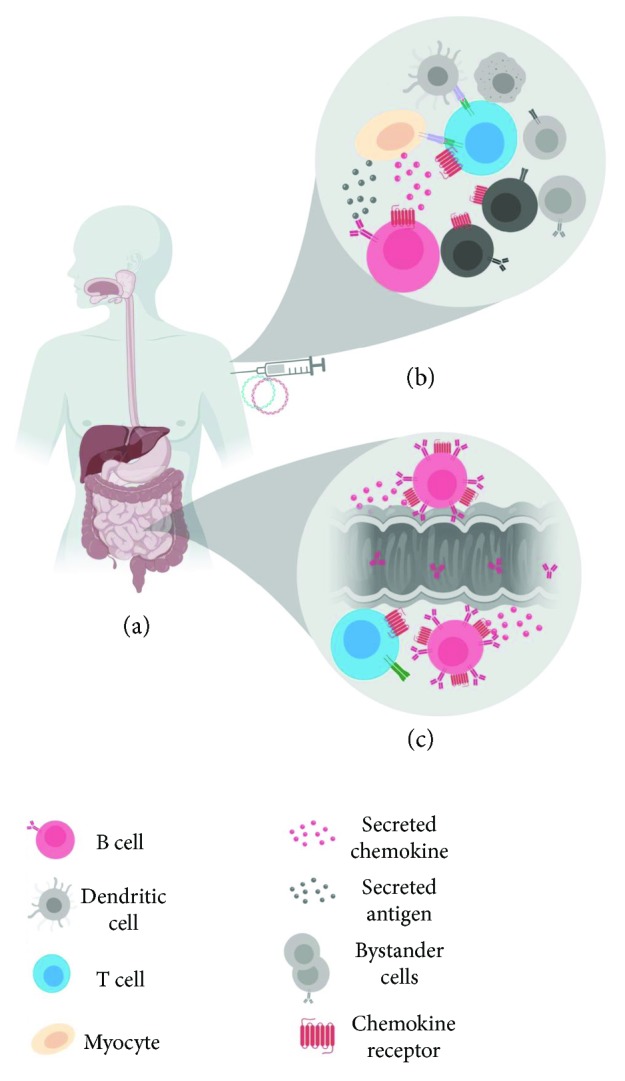
MALT molecular chemokine adjuvants in DNA vaccines. (a) Following parenteral delivery of antigen and chemokine plasmid DNA, transformed cells (tan) will transcribe, translate, process, and present the antigen. (b) Antigen (black circles) is also secreted from transformed cells, as is the chemokine adjuvant (red circles), creating a local chemokine gradient which will recruit chemokine receptor-bearing cells (red chemokine receptors). Some recruited cells will be unable to respond to antigen (dark gray); others will be recruited as a result of vaccination-induced inflammation (light gray). Recruited cells bearing the appropriate receptors and capable of responding to antigen (red B cell and blue T cell), will upregulate the chemokine receptor and proliferate. (c) Receptor upregulation following chemokine ligation and antigen-experience imprints these antigen-experienced cells with the ability to traffic to the MALT effector site, resulting in antigen-specific mucosal responses.

The prime and pull method effectively pulls antigen-primed cells to mucosal surfaces; however, the longevity of these responses needs to be explored. DNA vaccines are an established platform for the codelivery of molecular chemokine adjuvants. Interestingly, even though DNA vaccines are almost exclusively delivered parenterally, the inclusion of plasmid-encoded chemokines as molecular adjuvants enhances responses in the distal mucosa. We propose that ligation of chemokine with its cognate receptor creates an autocrine amplification loop that increases expression of the cognate receptor on the cell surface. This phenomenon polarizes the cell such that it is more responsive to the chemokine gradient. This suggests that transformed cells secreting the chemokine adjuvant create an artificial, temporary gradient which recruits receptor-bearing immunocytes and leads to upregulation of the chemokine receptor in question. If any of these immunocytes can respond to the antigen being secreted and presented by the transformed cell, they will become activated, traffic to draining lymph nodes, expand in population, and eventually home back to mucosal sites by virtue of enhanced mucosal chemokine receptor expression and the homeostatic gradient created by IECs (Figure 4—MALT molecular chemokine adjuvants in DNA vaccines). Very little is known about the regulation of chemokine receptor expression. It will be important to characterize cognate chemokine receptor expression on mucosal effector cells following vaccination with mucosal chemokine adjuvants; this knowledge will be critical to further development of chemokine adjuvants. Similarly, vaccination studies where chemokines are used as adjuvants should evaluate the effect of chemokine adjuvantation on receptor expression on antigen-specific and bystander cells.

Having determined that chemokines can be used effectively to enhance vaccine-mediated mucosal immunity, it will be important to study whether vaccination with these adjuvants induces the establishment of immune memory at mucosal sites. Furthermore, it is important to continue to study the basic mechanisms by which expression and kinetics of tissue-specific homing receptors are regulated. This knowledge will inform the development of other methods to promote receptor-ligand-mediated homing. For example, it is known that colonization of the intestines by commensal microbes promotes increased CCL28 secretion by intestinal epithelial cells [108].

In conclusion, an increased understanding of chemokine-mediated trafficking in the mucosa has prompted the use of these molecules as adjuvants to direct activated, antigen-experienced effector cells to mucosal surfaces. Chemokine molecular adjuvants, particularly CCL28, have proven especially useful for generating humoral anti-HIV immunity at mucosal sites, leading to protection from challenge in SIV models. DNA vaccines are well-suited for the delivery of chemokine adjuvants and represent a parenteral delivery method that can promote mucosal immunity. Thus, the combined use of the DNA platform and mucosal chemokine adjuvants has potential to induce robust anti-HIV responses in the mucosa and represents a new modality for generating antigen-specific mucosal immunity. The final challenge for successful delivery of chemokines as vaccine adjuvants is the generation of long-lived immunity at mucosal surfaces. Thus, future studies should address the ability of chemokines to promote mucosal memory in the context of vaccination.

## Figures and Tables

**Table 1:** HIV-1 vaccine studies using chemokine adjuvants. Italicized adjuvants are not discussed in this review.

Study (ref)	Antigen	Platform	Chemokine adjuvants	Target cells	Results
Kathuria et al. [103]	HIV gag and also influenza HA	DNA vaccine	CCL25	CTLs and CD4^+^ T cells	Increased IFN*γ*^+^ T cells in the spleen and MLN. Increased IgA^+^ ASCs in PPs and increased HIV-specific IgA in serum.

Hu et al. [105]	HIV gp140 (env)	DNA vaccine	CCL19, CCL28, and *also APRIL*		Enhanced HIV-specific serum and vaginal IgA. Neutralizing IgA in vaginal wash and increased CCR10^+^ B cells in MLNs.

Tregoning et al. [106]	HIV gp140 (env and trimeric)	Protein	CCL28 and *also TLR4L*		No increase in HIV-specific IgA in vaginal wash.

Kutzler et al. [107]	SIV gag, pol, env, and nef-rev	DNA vaccine	CCL25, CCL27, and CCL28		Increased HIV-specific IgG and IgA in serum and vaginal wash. Highest protection from SIV challenge (CCR10L group).

Song et al. [104]	HIV gag	DNA vaccine	CCL3, CCL19, and CCL20	CTLs and macrophages	Increased M*φ* recruitment and CTL activity.
